# Risk factors for the recurrence in pulmonary tuberculosis patients with massive hemoptysis

**DOI:** 10.1111/crj.13653

**Published:** 2023-07-05

**Authors:** Qiong Lin, Jian Chen, Tianxing Yu, Bing Gao, Kaijin Kuang, Yong Fan, Junping Xu, Xiaohua Li, Xin Lin, Liyu Xu

**Affiliations:** ^1^ Department of Respiratory Medicine Fuzhou No.1 Hospital Affiliated with Fujian Medical University Fuzhou Fujian China; ^2^ Department of Intervention Therapy Fuzhou No.1 Hospital Affiliated with Fujian Medical University Fuzhou Fujian China; ^3^ Department of Medical Imaging Fuzhou Second Hospital of Xiamen University (Fuzhou Second Hospital) Fuzhou Fujian China; ^4^ School of Finance Fujian Jiangxia University Fuzhou Fujian China; ^5^ Cent Lab Fuzhou No.1 Hospital Affiliated with Fujian Medical University Fuzhou Fujian China

**Keywords:** embolization, massive hemoptysis, prognosis, pulmonary tuberculosis

## Abstract

**Objectives:**

To evaluate the outcomes of bronchial artery embolization (BAE) for the treatment of massive hemoptysis in patients with pulmonary tuberculosis and identify risk factors that influence recurrence.

**Methods:**

A total of 81 patients with massive hemoptysis who underwent BAE between January 2014 and December 2017 were retrospectively reviewed. All of the patients had either a history of pulmonary tuberculosis or a current diagnosis of pulmonary tuberculosis. Follow‐up ranged from 18 to 66 months.

**Results:**

Hemoptysis was stopped or markedly decreased, with subsequent clinical improvement in 73 patients, while 11 patients experienced recurrence during the follow‐up period. Systemic‐pulmonary shunts and clinical failure showed a statistically significant correlation with the recurrence rate. The cumulative non‐recurrence rate was 95.3% for 3 months and 81.9% for more than 24 months. Complications were common (12.5%), but self‐limiting.

**Conclusions:**

BAE is a safe and effective treatment option for the control of massive hemoptysis in pulmonary tuberculosis patients. Systemic‐pulmonary shunts and clinical failure are the risk factors for recurrence.

## Graphical abstract

Bronchial artery embolization is a safe and effective treatment option for the control of massive hemoptysis in pulmonary tuberculosis patients.

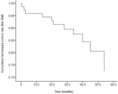

## 1 INTRODUCTION

Hemoptysis is one of the most frequent symptoms in patients with respiratory system diseases. In non‐western countries, pulmonary tuberculosis (TB) is still the most common cause of massive hemoptysis that may threaten the life of patients.^1^ Chronic inflammatory conditions of the lung such as TB may rupture and provide a significant source of hemoptysis, which has a variety of sequelae and complications such as fibrosis, cavities, aspergilloma, end‐stage lung destruction, airway lesions, and vascular lesions.

Generally, emergency treatment is performed to stop bleeding. However, the effects of conservative medical therapy are not quite satisfactory. The surgical excision has better effects, but the postoperative complications and mortality can be increased as well.^2^ With the recent development of interventional therapy, bronchial artery embolization (BAE) has been clinically proven to be effective for pulmonary TB patients with massive hemoptysis. After BAE, the recurrent bleeding rate is 10%–45%.^3^, ^4^, ^5^ Patients with chronic lung disease, including TB, have a particularly high recurrence rate. According to previous studies, recurrent bleeding may be caused by the recanalization of previously embolized arteries and/or the introduction of new bronchial and non‐bronchial arteries through disease progression and can be treated by repeated embolization or surgery.^4^, ^5^

Although BAE in TB patients has been studied previously, the results on risk factors for hemoptysis recurrence are not consistent. Therefore, this study aimed to evaluate the short‐term and long‐term effects of BAE and identify the risk factors for the recurrence in pulmonary TB patients with massive hemoptysis.

## 2 METHODS

### 2.1 Study population

This was a retrospective clinical study. In total, 81 patients with massive hemoptysis of TB or post‐TB sequelae in our hospital from January 2014 to December 2017 were reviewed. Massive hemoptysis was defined as (1) once expectoration of >100 mL blood or >500 mL within 24 h.^6^ All patients were treated with BAE for the first time. Patients who were unavailable for follow‐up were excluded. This study was approved by the Ethics Committee of Fujian Medical University Affiliated Fuzhou First Hospital, and all patients provided informed consent.

### 2.2 Clinical definitions

All of the patients were classified as follows^7^:

1. Active TB: no known history of TB and (i) AFB‐positive and/or images suggestive of active TB, or (ii) if AFB‐negative or unknown, current TB medication as the first treatment episode or images strongly suggestive of active TB and clinical suspicion.
2. TB sequelae: previous history of TB and AFB‐negative, with/without images suggestive of inactive TB.
3. TB reactivation: previous history of TB and AFB‐positive, with images suggestive of active/inactive TB.

According to the guidelines for percutaneous transcatheter embolization established by the Society of Interventional Radiology (SIR) Standards of Practice Committee,^8^ clinical success was defined as either complete stoppage of or reduced hemoptysis associated with a positive effect on clinical course. If hemoptysis continued without change, it was regarded as clinical failure. Recurrent hemoptysis was defined as the occurrence of frank hemoptysis with no blood‐tinged sputum, which should be treated with hospitalization or BAE.

### 2.3 Data collection

The general data on all patients were obtained from the standardized data sheet in the hospital's computer system, including demographic data, clinical manifestations, chest radiography, computed tomography (CT), and fiber optic bronchoscopy. The amount of bleeding and disease activity were measured. The outcomes of hemoptysis, postoperative complications, recurrent bleeding factors, and corresponding intervention measures were also recorded. For each patient, the end of follow‐up was defined as the date of death or the last day of March 2019. All patients were followed up by inpatient and outpatient medical records and by contact with the patient through telephone calls.

### 2.4 Management

All patients received conventional medical treatment after admission, including an unobstructed respiratory tract, oxygen uptake, hemostasis, symptomatic therapy, and anti‐tuberculous or antibiotics if necessary. The standard procedures of BAE were performed as follows: The modified Seldinger technique was used for femoral artery puncture; a 4F/5F catheter was placed for selective angiography. After the diagnosis, a 2.4 F microcatheter was inserted into the bronchial artery branch, and the coronary artery and spinal artery should be avoided in the process. According to the artery diameter and arteriovenous fistula, the materials for embolization were chosen, which were composed of gelfoam or gelfoam + coil.

### 2.5 Statistical analysis

Data were analyzed using SPSS v21.0 software (IBM Corp., Armonk, NY, USA). The Student's *t*‐test, Mann–Whitney U test, or Kruskal–Wallis test was used for continuous variables; the *χ* ^*2*^test or Fisher's exact test was used for categorical variables. The recurrent‐free time was calculated using the Kaplan–Meier method. Univariate Cox regression analysis was used for the univariate analysis of recurrence, and *p* < 0.1 was used for the screening of variables. The Cox proportional hazards model was used for multivariate analysis to identify the independent factors of recurrence‐free time. *p* < 0.05 was considered statistically significant.

## 3 RESULTS

### 3.1 Demographic characteristics and outcomes

A total of 81 patients underwent BAE procedures for massive hemoptysis due to TB or its sequelae. Hemoptysis was immediately stopped or markedly reduced, with subsequent improvement of the clinical course, in 73 patients (90.1%). Hemoptysis continued without change in eight patients after BAE; one patient died because of hemoptysis on the eighth day after BAE; others were improved after medical treatment or blood transfusion. Eight patients were lost to follow‐up after being discharged from the hospital. Eventually, 72 patients were involved in the analysis of recurrence outcomes.

**FIGURE 1:**
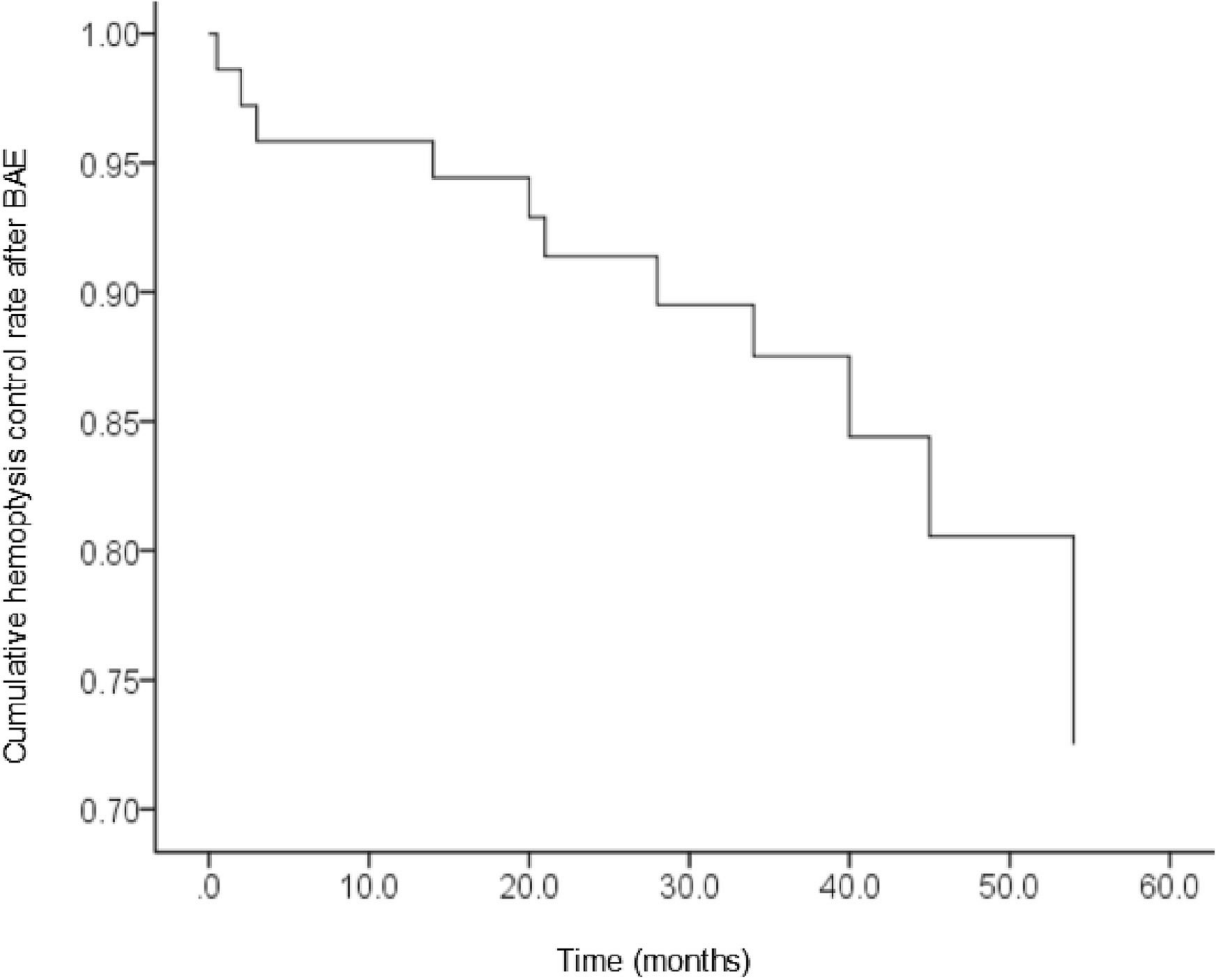
The cumulative hemoptysis control rate as depicted by the Kaplan–Meier method. BAE, bronchial artery embolization.

The 72 patients (61 males, 11 females) had a median age of 53 years old (18–86 years old) and a median bleeding volume of 600 mL (100–2000 mL). A total of 32 patients (44.4%) had active TB, including 8 cases of TB reactivation and 40 cases (55.6%) of TB sequelae (Table 1). The median follow‐up period was 37 months (range 18–61 months). A total of four patients died during the follow‐up period due to acute exacerbations of chronic bronchitis with respiratory failure (*n* = 1), pulmonary infection (*n* = 2), and lung cancer (*n* = 1), and none of them had hemoptysis recurrence. A total of 57 patients did not have recurrent hemoptysis, whereas 11 patients experienced recurrence. Among the 11 patients with recurrent hemoptysis, four patients improved upon conservative management, seven patients repeated BAE, and two cases underwent BAE for the third time (Tables 2 and 3). The cumulative hemoptysis control rate was depicted by the Kaplan–Meier curve (Figure 1).

**TABLE 1:** Demographic characteristics of 72 follow‐up patients.

Parameters	*N* (%) = 72
Gender	
Male	61 (84.722)
Female	11 (15.278)
Age, year	53 (18–86)
Disease course	
<1 month	48 (66.667)
>1 month	24 (33.333)
Amount of bleeding (mL)	600 (100–2000)
Diabetes mellitus	6 (8.333)
Disease activity	
TB sequelae	40 (55.556)
Active TB	24 (33.333)
TB reactivation	8 (11.111)

Abbreviation: TB, tuberculosis.

**TABLE 2:** The outcomes of 72 patients who underwent BAE for tuberculosis and post‐tuberculosis sequelae.

Outcome	*n*	Success	Failed BAE	Repeat BAE	Death	Successful repeat BAE	Cause of death	Recurrence factors
≤2 weeks	72	71	1	1	0	1	‐	Incomplete embolism
>2 weeks, ≤1 month	71	71	0	0	0	0	‐	‐
>1 month, ≤3 months	71	69	2	1	1	1	Acute exacerbation of chronic bronchitis with respiratory failure	Systemic‐pulmonary shunts
>3 months, ≤12 months	68	68	0	0	0	0	‐	‐
>12 months, ≤24 months	68	65	3	2	1	2	Pulmonary infection	Recanalization, systemic‐pulmonary shunts Recanalization
>24 months	64	59	5	4	2	3	Pulmonary infection Lung cancer	Progression of the underlying disease Recanalization Systemic‐pulmonary shunts Systemic‐pulmonary shunts

Abbreviation: BAE: bronchial artery embolization.

**TABLE 3:** List of 11 patients with delayed recurrence.

Patient no.	Follow‐up	Time of delayed recurrence	Treatment and outcome	Possible cause of recurrence
1	20 months	2 months 18 months	BAE, good BAE, good	Bronchoarteriopulmonary fistula
2	37 months	20 months 31 months	BAE, good BAE, good	Bronchoarteriopulmonary fistula
3	24 months	3 months 6 months	Drug, good BAE, good	Bronchoarteriopulmonary fistula
4	50 months	34 months	BAE, good	New lesion bleeding
5	31 months	21 months	BAE, good	Recanalization after BAE
6	28 months	0.5 month	BAE, good	Incomplete embolization
7	57 months	54 months	BAE, good	Bronchoarteriopulmonary fistula
8	45 months	44 months	BAE, good	Recanalization after BAE
9	53 months	14 months	Drug, good	Right superior bronchial artery juxtaposed with orifice of intercostal artery
10	54 months	27 months	Drug, good	Large lung lesions with abundant branches
11	53 months	37 months	Drug, good	Bronchoarteriopulmonary fistula

Abbreviation: BAE: bronchial artery embolization.

### 3.2 Factors affecting outcome of BAE

Of the 72 patients, 11 (15.3%) experienced hemoptysis recurrence during the follow‐up period. Table 4 summarizes the results of the univariate Cox regression analysis. For clinical factors, outcome after BAE demonstrated statistical significance with recurrence (odds ratio [OR], 0.242; 95% confidence interval [CI], 0.064–0.914; *p* = 0.013). For image and angiographic factors, systemic‐pulmonary shunts were present in 14 cases (19.444%), which was highly correlated with recurrence (OR, 3.593; 95% CI, 1.094–11.800; *p* = 0.035). Aspergilloma, which was performed in 15 patients (20.833%), also showed statistical significance with regard to recurrence (OR, 3.829; 95% CI, 1.156–12.688; *p* = 0.028).

**TABLE 4:** Univariate Cox regression analysis of risk factors affecting rebleeding after BAE.

Etiology	Case (*n* = 72) *n* (%)	Recurrent (*n* = 11) *n* (%)	Non‐recurrent (*n* = 61) *n* (%)	*p* value	Odds ratio (95% CI)
Gender					
Male	61 (84.722)	9 (81.818)	52 (85.246)	0.936	1.065 (0.227–4.998)
Female	11 (15.278)	2 (18.182)	9 (14.754)		
Amount of bleeding (mL)	600 (100–2000)	600 (100–2000)	500 (100–1600)	0.732	1 (0.998–1.001)
Diabetes mellitus	6 (8.333)	1 (9.091)	5 (8.197)	0.609	1.718 (0.216–13.678)
Outcome after BAE					
Clinical success	66 (91.667)	8 (72.727)	58 (95.082)	0.013	0.242 (0.064–0.914)
Clinical failure	6 (8.333)	3 (27.273)	3 (4.918)		
Disease activity					
TB sequelae	40 (55.556)	9 (81.818)	31 (50.820)	0.066	4.288 (0.910–19.657)
Active tuberculosis	32 (44.444)	2 (18.182)	30 (49.180)		
Systemic‐pulmonary shunts	14 (19.444)	5 (45.455)	9 (14.754)	0.035	3.593 (1.094–11.800)
Embolism materials					
Gelfoam	25 (34.722)	4 (36.364)	21 (34.426)	0.564	0.681 (0.185–2.511)
Gelfoam + coil	47 (65.278)	7 (63.636)	40(65.574)		
CT findings					
Fibrotic scar change	51 (70.833)	6 (54.545)	45 (73.770)	0.156	0.421 (0.127–1.390)
Cavity	32 (44.444)	3 (27.273)	29 (47.541)	0.202	0.420 (0.111–1.591)
Bronchiectasis	39 (54.167)	9 (81.818)	30 (49.180)	0.102	3.592 (0.775–16.652)
Aspergilloma	15 (20.833)	5 (45.455)	10 (16.393)	0.028	3.829 (1.156–12.688)
TB destroyed lung	15 (20.833)	3 (27.273)	12 (19.672)	0.609	1.417 (0.373–5.386)

Abbreviations: BAE, bronchial artery embolization; TB, tuberculosis.

Neither of the groups showed statistically significant differences with regard to the following: the presence of TB sequelae such as 11.8% (6 of 51) for fibrotic scar change, 9.4% (3 of 32) for cavity, 23.1% (9 of 39) for bronchiectasis, and 20.0% (3 of 15) for TB‐destroyed lung. Disease activity was not associated with the risk of rebleeding (OR, 4.288; 95% CI, 0.910–19.657; *p* = 0.066). Embolism materials, sex, amount of bleeding, or diabetes mellitus were not associated with the risk of recurrence.

**FIGURE 2:**
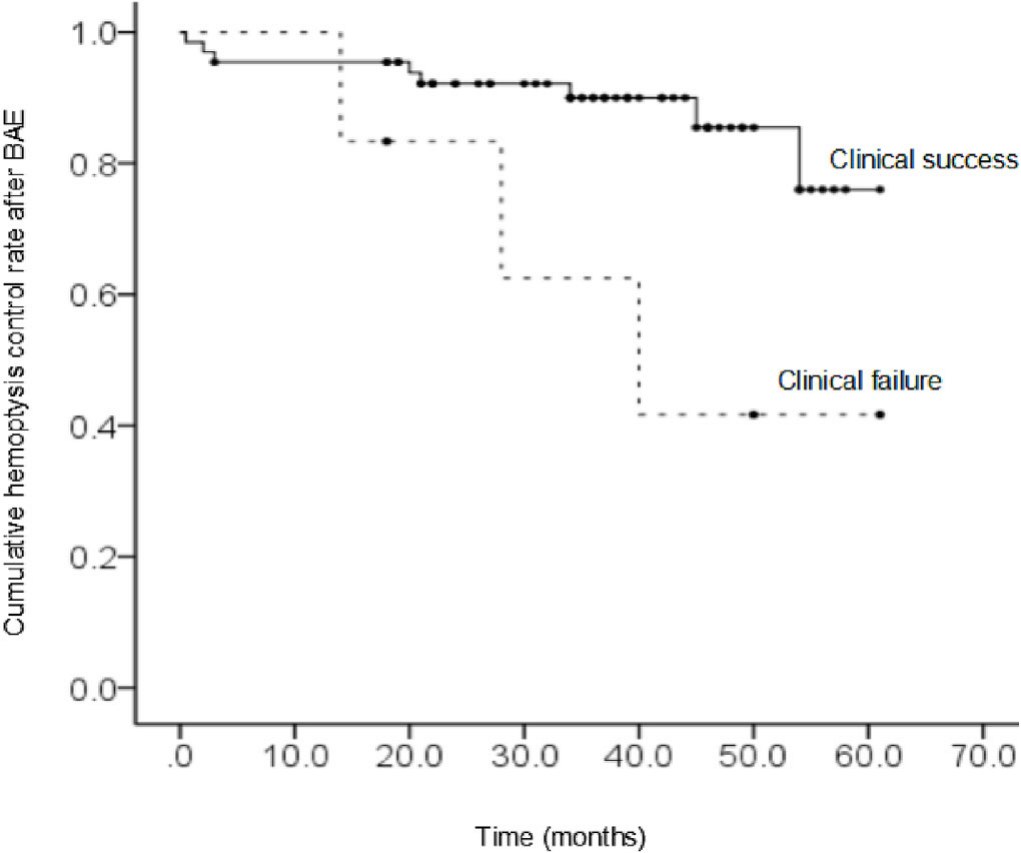
The cumulative hemoptysis control rate as depicted by the Kaplan–Meier method: *p* = 0.013; clinical success versus clinical failure. BAE, bronchial artery embolization.

**FIGURE 3:**
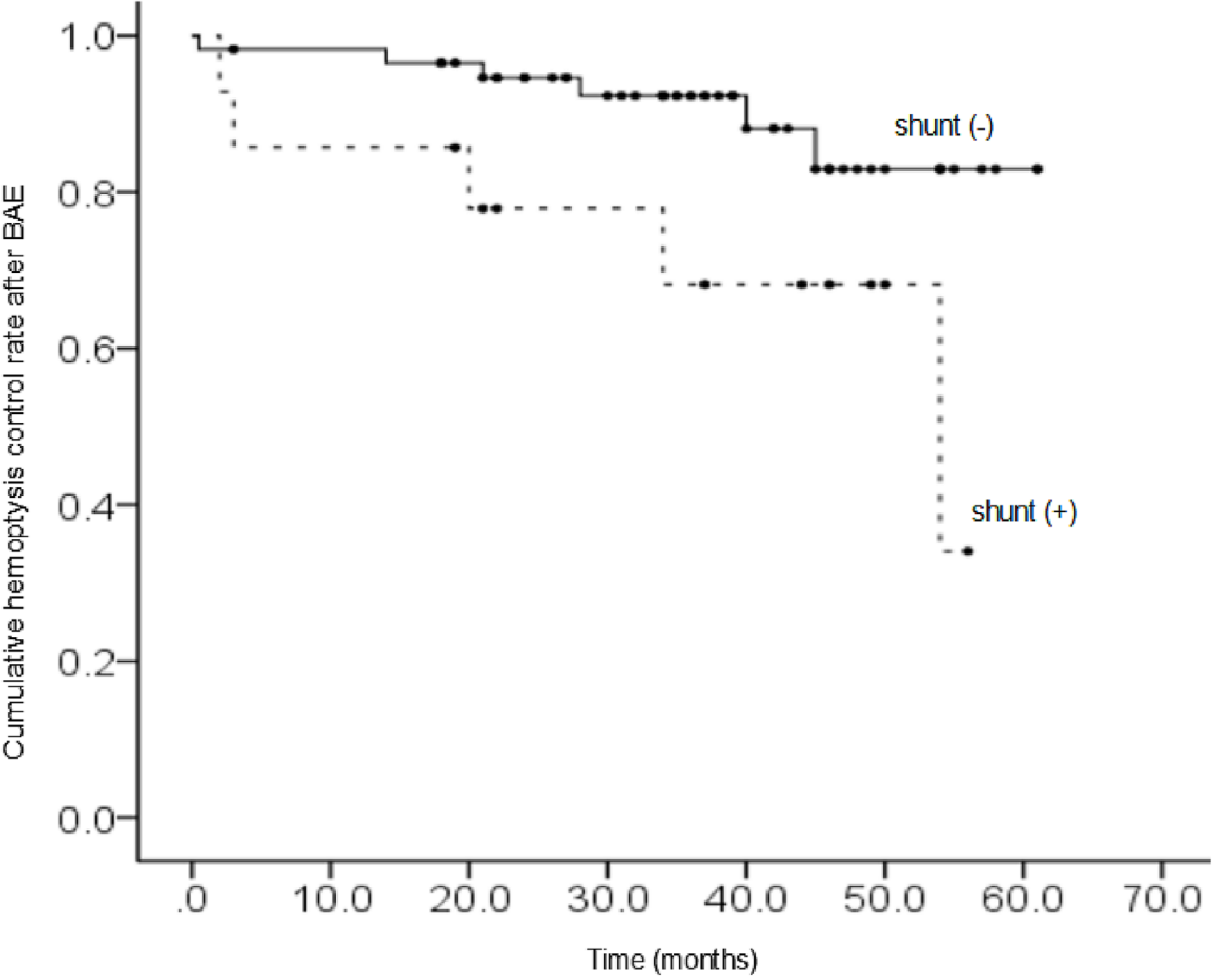
The cumulative hemoptysis control rate as depicted by the Kaplan–Meier method: *p* = 0.035; shunt (−) versus shunt (+). BAE, bronchial artery embolization.

Table 5 shows the result of the multivariate Cox regression analysis. The outcome after BAE and systemic‐pulmonary shunts showed statistical significance. The cumulative hemoptysis control rates were depicted by the Kaplan–Meier curve for the outcome after BAE and systemic‐pulmonary shunts, respectively (Figures 2 and 3).

**TABLE 5:** Multivariate Cox regression analysis of the factors of recurrence‐free time after BAE.

	*p* value	Odds ratio (95% CI)
Systemic‐pulmonary shunts	0.024	5.303(1.242–22.640)
Outcome after BAE	0.034	0.168(0.032–0.878)
Aspergilloma	0.280	2.096(0.547–8.036)

Abbreviation: BAE, bronchial artery embolization.

### 3.3 Complications

A total of 9 patients experienced procedure‐related complications (12.5%), including gastrointestinal symptoms (*n* = 3), fever (*n* = 4), chest tightness (*n* = 1), and shortness of breath (*n* = 1). Only one patient died after intervention; the cause of his hemoptysis was secondary pulmonary TB, and the cause of his death was massive hemoptysis asphyxia. Most procedure‐related complications were minor and could be improved spontaneously or by nominal therapy. No serious complications, such as a spinal artery embolism, occurred.

## 4 DISCUSSION

Massive hemoptysis is one of the most common complications in pulmonary TB patients, which may lead to respiratory tract obstruction and asphyxia, hemorrhagic shock, or even death. Meanwhile, patients could suffer heavy psychological burdens due to aggravated illness states and the difficulties of medical treatment, which affect the final treatment outcomes.^9^, ^10^ Previous reports showed that the mortality rate for pulmonary TB patients with massive hemoptysis could be as high as 50% without standardized treatment.^11^ Although the pulmonary lobectomy has shown good effects, it is limited due to severe trauma, which may affect the life quality, functional status, and complications of the patients. However, conservative medical therapy extends the treatment time and produces side effects, and patients are prone to have palpitations, emesis, and nausea while applying pituitrin for vasoconstriction in the long term.^12^ With recent technological development, vascular interventional therapy has made great achievements. Interventional therapy has been primarily recommended for favorable therapeutic effects in pulmonary TB with massive hemoptysis.^13^

Previous studies had shown that the treatment efficacy of bronchial arterial embolization in patients with massive hemoptysis of TB was as high as 90%.^14^, ^15^, ^16^ Our immediate success rate of BAE was 90.1%, which was consistent with previous studies. During the follow‐up period, the recurrence rate was 15.3% in this study, which was comparable to previous reports ranging from 10% to 45%.^5^, ^17^

There are two peak periods of hemoptysis recurrence. The first peak for recurrence is from 1 to 2 months after BAE, which may be because of incomplete embolism. The second peak for recurrence is from 1 to 2 years after BAE. This seems to reflect the recruitment of blood supply and revascularization due to the progression of the underlying disease. No similar peak recurrence period was observed in our study. Consistent with previous studies, short‐term bleeding recurrence reflects incomplete embolism, whereas long‐term bleeding recurrence may be probably because of recanalization after BAE, systemic‐pulmonary shunts, and the progression of the underlying disease.^18^ Overall, BAE has a high success rate in treating massive hemoptysis of TB, which can be recognized as the first‐line treatment for replacement of high‐risk emergency surgery or selective surgery. Moreover, for massive hemoptysis caused by TB, the outcome of repeated BAE procedures is generally good.

High recurrence rates for patients with aspergilloma have been shown in previous reports.^19^, ^20^ Patients with aspergilloma have extensive parasite circulation from different sources, which may be associated with vasculitis in cases of thick vascular cavitary wall. In our study, aspergilloma was associated with the rate of recurrent hemoptysis in univariate Cox regression analysis. However, there was no significant difference in multivariate Cox regression. The sample size might be too small to detect the difference between the two groups.

Lee et al.^17^ reported that active TB was related to the recurrence rate after BAE. Active TB patients have persistent active mucosal inflammation, which is regarded as a recurrence factor after BAE. However, other studies showed that active TB was not related to the recurrence rate after BAE.^21^, ^22^, ^23^ Some studies have shown that active TB had more favorable results in terms of recurrence. In our study, we found no significant effects of TB sequelae and active TB on recurrence.

Our analysis showed that systemic‐pulmonary shunts and clinical failure were risk factors for the recurrence of BAE in patients with tuberculous hemoptysis. Especially with the increased shunt, the large size of embolic materials should be chosen to prevent emboli from entering into the pulmonary circulation. As a consequence, the incomplete blood vessel embolism leads to recurrent hemoptysis.^9^, ^24^, ^25^, ^26^ Clinical failure is an important factor for the risk of recurrence after BAE because patients who fail BAE for the first time will have a higher recurrence rate in the future. It might be postulated that surgical treatment should be recommended for these patients.

In this study, the complication rate was 11.1% (9/81). All of the symptoms were mild and could be managed conservatively. Other major complications, such as spinal cord ischemia or mediastinal structure necrosis, did not occur. Procedure‐related complications are common, but most are minor.^27^ This is probably contributed to the use of digital subtraction angiography (DSA), super‐selective catheterization of the abnormal vessel, and the use of a micro‐catheter for the particle embolization.

Our study has some limitations. First, this is a single‐center study, and the sample size might not be sufficient to identify minor factors that could be related to the recurrence of hemoptysis. Second, the study design is retrospective, which may cause high risk of biases.

In conclusion, BAE is a safe and effective procedure with minimal complications that can be performed routinely in pulmonary TB patients presenting with massive hemoptysis. Systemic‐pulmonary shunts and clinical failure are the risk factors for recurrence.

## AUTHOR CONTRIBUTIONS

Qiong Lin, Jian Chen, Tianxing Yu, Bing Gao, Kaijin Kuang, Yong Fan, Junping Xu, Xiaohua Li, and Xin Lin collected and analyzed the data, Liyu Xu designed the study and wrote the manuscript. All authors read and approved the manuscript.

## CONFLICT OF INTEREST STATEMENT

The authors declare that they have no competing interests.

## ETHICS STATEMENT

This study was approved by the Ethics Committee of Fujian Medical University Affiliated Fuzhou First Hospital, and all patients provided informed consent.

## Data Availability

All data are available from the corresponding author upon reasonable request.

## References

[1] Kalva SP . Bronchial artery embolization. Tech Vasc Interv Radiol. 2009;12(2):130‐138. doi:10.1053/j.tvir.2009.08.006 19853230

[2] Wang SM , Yang S . Study the etiology of life‐threatening massive haemoptysis and the efficacy of selective bronchial artery embolization. J Clin Pulm Med. 2016;21(10):1791‐1794.

[3] Racil H , Rajhi H , Ben Naceur R , Chabbou A , Bouecha H , Mnif N . Endovascular treatment of haemoptysis: medium and long‐term assessment. Diagn Interv Imaging. 2013;94(1):38‐44. doi:10.1016/j.diii.2012.05.010 23246187

[4] Garcia‐Olive I , Sanz‐Santos J , Centeno C , et al. Predictors of recanalization in patients with life‐threatening hemoptysis requiring artery embolization. Arch Bronconeumol. 2014;50(2):51‐56. doi:10.1016/j.arbr.2014.01.004 23932187

[5] Kim YG , Yoon HK , Ko GY , et al. Long‐term effect of bronchial artery embolization in Korean patients with haemoptysis. Respirology (Carlton, Vic). 2006;11(6):776‐781. doi:10.1111/j.1440-1843.2006.00946.x 17052307

[6] Dweik RA , Stoller JK . Role of bronchoscopy in massive hemoptysis. Clin Chest Med. 1999;20(1):89‐105. doi:10.1016/S0272-5231(05)70129-5 10205720

[7] Chen BR , Zhou D , Nie L , et al. Retrospective analysis in 63 bronchiectasia patients with massive haemoptysis underwent bronchial artery embolization. Shanxi Med J. 2017;46(16):2001‐2003.

[8] Drooz AT , Lewis CA , Allen TE , et al. Quality improvement guidelines for percutaneous transcatheter embolization. Journal of Vascular and Interventional Radiology: JVIR. 2003;14(9 Pt 2):S237‐S242. 14514825

[9] Kokkonouzis I , Athanasopoulos I , Doulgerakis N , et al. Fatal hemoptysis due to chronic cavitary pulmonary aspergillosis complicated by nontuberculous mycobacterial tuberculosis. Case Reports in Infectious Diseases. 2011;2011:1‐4. doi:10.1155/2011/837146 PMC3336246 22567480

[10] Kawami M , Yumoto R , Takano M . Preventive approach against drug‐induced pulmonary fibrosis through the suppression of epithelial‐mesenchymal transition. Biocell. 2022;46(8):1861‐1865. doi:10.32604/biocell.2022.019667

[11] Wu CM , Qiu YL , Yin JT , et al. Clinical retrospective analysis in 148 massive haemoptysis patients of undergoing bronchial artery embolization. Int J Respir. 2017;37(2):130‐135.

[12] Prasad R , Garg R , Singhal S , Srivastava P . Lessons from patients with hemoptysis attending a chest clinic in India. Ann Thor Med. 2009;4(1):10‐12. doi:10.4103/1817-1737.43062 PMC2700474 19561915

[13] Kwon W , Kim YJ , Lee YH , Lee WY , Kim MS . The effectiveness of embolotherapy for treatment of hemoptysis in patients with varying severity of tuberculosis by assessment of chest radiography. Yonsei Med J. 2006;47(3):377‐383. doi:10.3349/ymj.2006.47.3.377 16807988 PMC2688158

[14] Shin BS , Jeon GS , Lee SA , Park MH . Bronchial artery embolisation for the management of haemoptysis in patients with pulmonary tuberculosis. Int J Tuberc Lung Dis. 2011;15(8):1093‐1098. doi:10.5588/ijtld.10.0659 21740674

[15] Xu W , Wang HH , Bai B . Emergency transcatheter arterial embolization for massive hemoptysis due to pulmonary tuberculosis and tuberculosis sequelae. Cell Biochem Biophys. 2015;71(1):179‐187. doi:10.1007/s12013-014-0182-3 25134662

[16] Anuradha C , Shyamkumar NK , Vinu M , Babu NR , Christopher DJ . Outcomes of bronchial artery embolization for life‐threatening hemoptysis due to tuberculosis and post‐tuberculosis sequelae. Diagn Interv Radiol (Ankara, Turkey). 2012;18(1):96‐101. doi:10.4261/1305-3825.DIR.3876-11.2 21678246

[17] Lee S , Chan JW , Chan SC , et al. Bronchial artery embolisation can be equally safe and effective in the management of chronic recurrent haemoptysis. Hong Kong Med J = Xianggang Yi Xue Za Zhi. 2008;14(1):14‐20. 18239238

[18] Ryuge M , Hara M . Mechanisms of recurrent haemoptysis after super‐selective bronchial artery coil embolisation: a single‐centre retrospective observational study. Eur Radiol. 2019;29(2):707‐715. doi:10.1007/s00330-018-5637-2 30054792 PMC6302874

[19] Hwang HG , Lee HS , Choi JS , Seo KH , Kim YH , Na JO . Risk factors influencing rebleeding after bronchial artery embolization on the management of hemoptysis associated with pulmonary tuberculosis. Tuberculosis and Respiratory Diseases. 2013;74(3):111‐119. doi:10.4046/trd.2013.74.3.111 23579345 PMC3617130

[20] Peng Y , Zhu Y , Ao G , et al. Effect of bronchial artery embolisation on the management of tuberculosis‐related haemoptysis. The International Journal of Tuberculosis and Lung Disease: the Official Journal of the International Union against Tuberculosis and Lung Disease. 2019;23(12):1269‐1276. doi:10.5588/ijtld.19.0135 31931910

[21] Van den Heuvel MM , Els Z , Koegelenberg CF , et al. Risk factors for recurrence of haemoptysis following bronchial artery embolisation for life‐threatening haemoptysis. Int J Tuberc Lung Dis. 2007;11(8):909‐914. 17705959

[22] Chun JY , Belli AM . Immediate and long‐term outcomes of bronchial and non‐bronchial systemic artery embolisation for the management of haemoptysis. Eur Radiol. 2010;20(3):558‐565. doi:10.1007/s00330-009-1591-3 19727742

[23] Kato A , Kudo S , Matsumoto K , et al. Bronchial artery embolization for hemoptysis due to benign diseases: immediate and long‐term results. Cardiovasc Intervent Radiol. 2000;23(5):351‐357. doi:10.1007/s002700010062 11060364

[24] Karmakar S , Nath A , Neyaz Z , Lal H , Phadke RV . Bronchial artery aneurysm due to pulmonary tuberculosis: detection with multidetector computed tomographic angiography. J Clin Imaging Sci. 2011;1:26. doi:10.4103/2156-7514.81293 21966623 PMC3177409

[25] Yoon JY , Jeon EY , Lee IJ , Koh SH . Coronary to bronchial artery fistula causing massive hemoptysis in patients with longstanding pulmonary tuberculosis. Korean J Radiol. 2012;13(1):102‐106. doi:10.3348/kjr.2012.13.1.102 22247644 PMC3253394

[26] Lu GD , Zu QQ , Zhang JX , et al. Risk factors contributing to early and late recurrence of haemoptysis after bronchial artery embolisation. Int J Tuberc Lung Dis. 2018;22(2):230‐235. doi:10.5588/ijtld.17.0543 29506621

[27] Springer DM , Cofta S , Juszkat R , et al. The effectiveness of bronchial artery embolisation in patients with haemoptysis. Adv Respir Med. 2018;86(5):220‐226. doi:10.5603/ARM.2018.0035 30378649

